# A tale of two domains: how PIF7 controls hypocotyl growth

**DOI:** 10.1093/plphys/kiag729

**Published:** 2026-09-30

**Authors:** Hannah Rae Thomas

**Affiliations:** Assistant Features Editor, Plant Physiology, American Society of Plant Biologists, Rockville, United States; Department of Horticulture, Zijingang Campus, Zhejiang University, 866 Yuhangtang Road, Hangzhou 310058, PR China

In addition to its influence on photosynthesis, light acts as a power signal for plants. One major input plants detect is the ratio of red (R) to far-red (FR) light. Sunny days have a high R:FR ratio, while shade from cloud cover or nearby vegetation is enriched for FR light, leading to low R:FR perception. Light-sensing PHYTOCHROME (Phy) A and B act to trigger downstream responses to either high or low R:FR (Smith and Whitelam 1997). PhyB is critical for shade avoidance syndrome (SAS), which occurs when high levels of FR (low R:FR) are detected, shifting the light sensor from its active (Pfr) state to the inactive state (Pr) (Leivar et al. 2008). In low R:FR light, SAS-promoting transcription factors (TFs), such as PHYTOCHROME INTERACTING FACTORS (PIFs), bind to PIF-binding E-box (PBE) motifs in the promoters of various shade-responsive genes, activating various processes such as auxin biosynthesis, hypocotyl and petiole elongation, and leaf hyponasty (Roig-Villanova et al. 2007; Lorrain et al. 2008; Hornitschek et al. 2012; Hussain et al. 2022). In high R light (high R:FR), PhyB binds to PIFs, rapidly leading to their phosphorylation and degradation, making PIF activity highly sensitive to changes in light conditions (Park et al. 2004; Al-Sady et al. 2006; Leivar and Quail 2011). This PhyB-PIF interaction occurs at the PIF Active Phytochrome B-binding (APB) region (Shen et al. 2008). In PIF3, the APB is required for hypocotyl elongation but not for early gene expression changes, suggesting that PIF3's full activity may be more complex than acting solely as a TF (Al-Sady et al. 2006).

In contrast to PIF1, 3, 4, and 5, PIF7 is relatively photostable and has been shown to be involved in shade-induced hypocotyl elongation (Lorrain et al. 2008; Li et al. 2012). Yet the exact molecular mechanism of PIF7 activity is not fully understood, especially the somewhat separate interactions between active PhyB-PIF7 through the APB and PIF7-DNA via the bHLH PBE. Recently, in *Plant Physiology*, Diaz-López et al. (2026) explored the exact role of PhyB-PIF7 in shade-induced hypocotyl elongation and gene expression and how this function was retained throughout dicot evolution.

To first look at the conserved role of PIF7, the authors generated *slpif7ab* and *chpif7* mutants in shade-avoidant *Solanum lycopersicum* (tomato) and shade-tolerant *Cardamine hirsuta* (Hairy bittercress). Using these mutant lines, they showed that in tomato, loss of *SlPIF7a/b* reduced seedling length under both white light (W) and simulated shade (W + FR) without eliminating the relative elongation response to shade. In *C. hirsuta*, ChPIF7 promoted growth under both conditions, while its contribution to shade-induced elongation became evident only in the phyA-deficient *sis1* background. They concluded that although shade-tolerant and shade-avoidant plants respond significantly differently to low R:FR, they retain some conserved PIF7 functions.

Next, to tease apart the role of PhyB and the bHLH domain on PIF7's biological activity versus the role of PIF7 as a gene regulator, they introduced 3 PIF7 variants into the *Atpif7* mutant: the wild-type PIF7 coding sequence (*pif7^PIF7WT^*), PIF7 with a mutated APB site (*pif7^PIF7APBm^*), and PIF7 with a mutated bHLH region required for DNA binding (*pif7^PIF7Bm^*), all under the control of a native PIF7 promoter fragment (Fig. 1). When examining the effect of these derivatives on hypocotyl elongation, they found that *pif7^PIF7Bm^* was as stunted as the *pif7* mutant, suggesting it had no biological activity, while *pif7^PIF7APBm^* showed significantly longer hypocotyls than *pif7^PIF7WT^* in W light, suggesting constitutive PIF7 activity. When treated with W + FR, *pif7^PIF7WT^* and *pif7^PIF7APBm^* mutants elongated, while *pif7^PIF7Bm^* did not. However, when comparing W and W + FR, only *pif7^PIF7WT^* showed a significant shade-induced increase. This showed that the bHLH region is critical for PIF7's function in hypocotyl elongation but that PhyB interaction with PIF7 is necessary for shade-induced hypocotyl elongation.

To explore the cause of the *pif7^PIF7APBm^* elongated hypocotyl, they quantified the amount of active/inactive PIF7 present in the 3 derivatives and found that while *pif7^PIF7WT^* and *pif7^PIF7Bm^* both experienced a dynamic increase in active PIF7 in W + FR and a decrease in W, *pif7^PIF7APBm^* contained constitutively high levels of active dephosphorylated PIF7, which suggests that the interaction with PhyB is also required for PIF7 phosphorylation regulation.

After exploring the PhyB-PIF7 interaction in hypocotyl elongation, they next set out to determine PIF7's role as a transcriptional regulator. To do this, they examined the known PIF7 target genes *PIL1*, *YUC8*, and *YUC9*. The *pif7^PIF7Bm^* variant showed expression similar to *pif7*, consistent with a complete loss of DNA-binding capacity due to mutation to the bHLH dimerization and DNA-binding domain. In contrast, *pif7^PIF7APBm^* showed a constitutive expression pattern, independent of shade treatment. They were then able to show via ChIP-qPCR that the high levels of *PIL1* expression in *pif7^PIF7APBm^* were due to PIF7 directly binding to the E-box, regardless of light condition.

The study shows that PhyB binding through the APB motif makes PIF7 activity responsive to light conditions by regulating its abundance and phosphorylation state. In contrast to PIF3, PIF7's DNA-binding activity is essential for both target-gene regulation and hypocotyl elongation.

However, a few questions remain. In this work, the authors could not fully complement the *pif7* mutant with *pif7^PIF7WT^.* They hypothesize that this could be due to the 2-kb promoter's inability to confer precise gene regulation or the lack of introns in the subsequent transgenes, but it is also possible that protein levels differed in the *pif7^PIF7WT^* derivative compared with true WT despite using lines with similar transcript abundance. Last, the authors encountered some phenotype discrepancies in their analysis of *PIF7* in tomato. Previous work has shown that PIF7 did not regulate shade-induced hypocotyl elongation, with *slpif4slpif7aslpif7b* elongating similarly to the M82 wild-type background (Kunta et al. 2025). However, this work used the dwarfing Microtom tomato variety and found that *slpif7aslpif7b* showed a significantly reduced elongation phenotype; however, it lacked a shade-induced phenotype. The authors noted this discrepancy and argued that, in tomato, different backgrounds appear to behave differently, clouding any conclusions about high levels of genetic conservation. Regardless, their ability to dissect activation from gene regulation in *PIF7* shows that the balance between positive and negative regulation is key to hypocotyl elongation and SAS.

**Figure 1:**
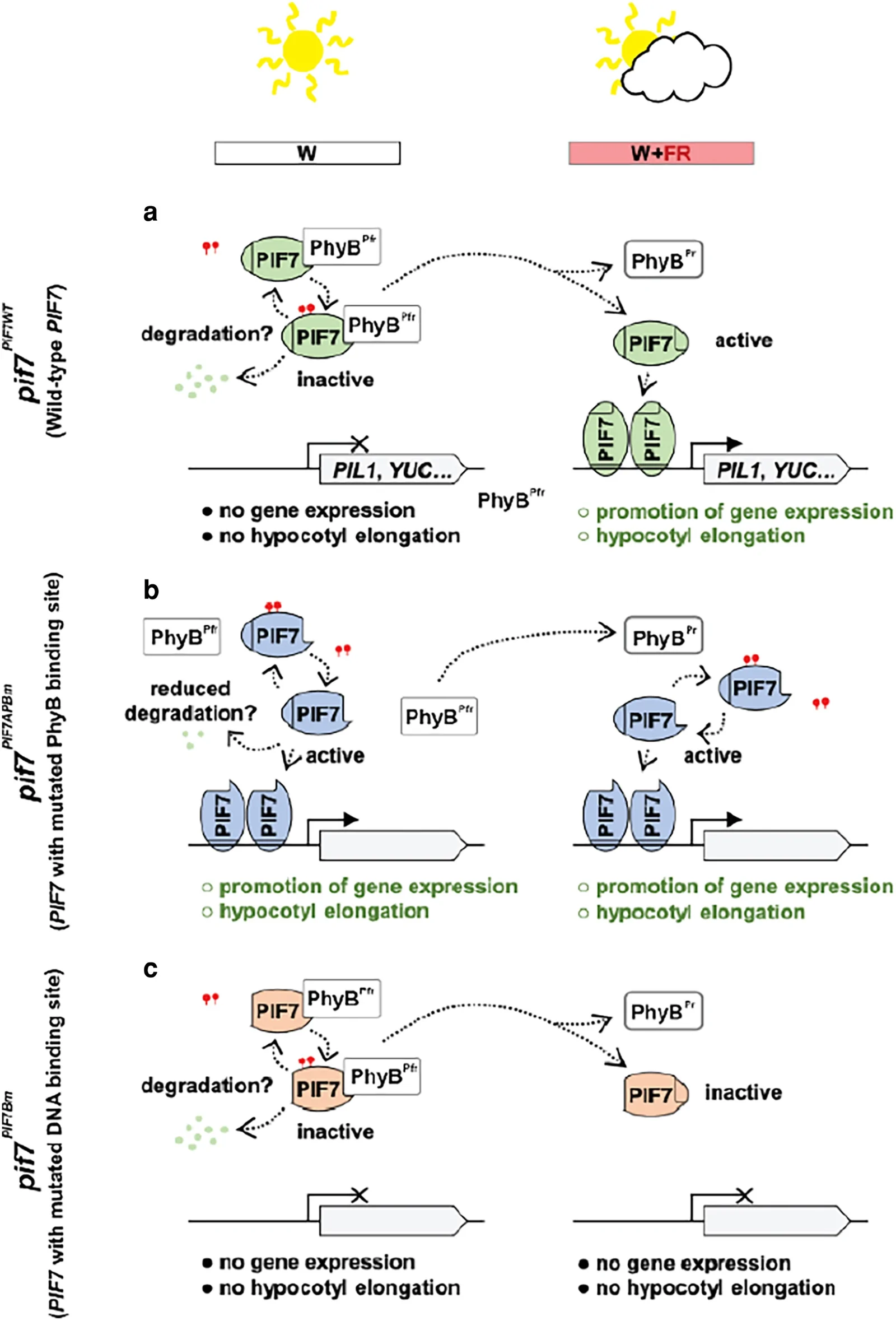
PIF7 derivatives elucidate the roles of PhyB and DNA binding on biological activity. In both wild-type (WT) and WT-complemented *pif7* mutants (*pif7^PIF7WT^*), white (W) light activates PhyB (Pfr), which binds PIF7, maintaining its inactive phosphorylated state (red dots) and repressing its activity. During simulated shade (W + far-red(FR)), PhyB is inactivated (Pr), releasing PIF7, which allows it to dephosphorylate and act as a transcriptional regulator and promote hypocotyl elongation. Complementation of the *pif7* mutant with a *PIF7* gene lacking the PhyB binding site (*pif7^PIF7APBm^)* led to constitutive activation of PIF7 due to the inability of PhyB to repress its activity in W light. In contrast, complementation of the *pif7* mutant with a *PIF7* gene lacking the DNA-binding site (*pif7^PIF7Bm^*) led to a complete lack of PIF7 activity regardless of light conditions. Shade is depicted as cloud cover, but it can also be caused by vegetation proximity. Adapted from (Diaz-López et al. 2026).

**Recent related articles in *Plant Physiology*:**

Srinivas Kunta et al. Phytochrome-interacting factors integrate environmental signals to regulate tomato growth and development, *Plant Physiology*, Volume 201, Issue 3, July 2026, kiag379, https://doi.org/10.1093/plphys/kiag379.
Matías Ezequiel Pereyra et al. Avoiding the shadow: how plants perceive neighbors and reshape the crop light environment, *Plant Physiology*, Volume 200, Issue 2, February 2026, kiag034, https://doi.org/10.1093/plphys/kiag034.
Silpa M G et al. Decoding light perception and fine-tuning photomorphogenesis through post-translational modifications, *Plant Physiology*, 2026; kiag189, https://doi.org/10.1093/plphys/kiag189.

## Data Availability

Not applicable.
